# Adamantinoma: A clinicopathological review and update

**DOI:** 10.1186/1746-1596-3-8

**Published:** 2008-02-15

**Authors:** Deepali Jain, Vijay K Jain, Rakesh K Vasishta, Prabhat Ranjan, Yashwant Kumar

**Affiliations:** 1 Department of Pathology, Maulana Azad Medical College New Delhi, India; 2 Department of Orthopedics Dr Ram Manohar Lohia Hospital, New Delhi, India; 3 Department of Histopathology, Post Graduate Institute of Medical Education and Research, Chandigarh, India

## Abstract

Adamantinoma is a primary low-grade, malignant bone tumor that is predominantly located in the mid-portion of the tibia. The etiology of the tumor is still a matter of debate. The initial symptoms of adamantinoma are often indolent and nonspecific and depend on location and extent of the disease. Histologically, classic adamantinoma is a biphasic tumor characterized by epithelial and osteofibrous components that may be intermingled with each other in various proportions and differentiating patterns. To assure the histological diagnosis, pathologists should employ immunohistochemistry for demonstrating the sometimes sparse epithelial cell nests when the radiological features are suggestive of adamantinoma. There is paucity of compiled data over adamantinoma in the literature, hence authors tried to make a comprehensive review which must be of use to beginners and trained pathologists. Our objective is to further define the clinicoradiologic features and pathologic spectra of adamantinoma.

## Background

Adamantinoma is a primary low-grade, malignant bone tumor, of unknown histogenesis. However recent opinion seems to suggest that adamantinoma is a tumor of epithelial origin, based on ultrastructural and immunohistochemical studies [1]. The tumor is located predominantly in the mid-portion of the tibia. The cases do occur simultaneously in tibia and in adjacent fibula or may occur in fibula with out involvement of tibia. It is a rare neoplasm, comprise only 0.1–0.5% of all primary bone tumors [2]. The first reported example is attributed to Maier in 1900 [3]. In 1913, Fischer [4] named the lesion "primary adamantinoma of the tibia" because of its striking histologic resemblance to the jaw "adamantinoma" (ameloblastoma). In 1951, Schulenberg [5] suggested a unifying histogenetic concept for the adamantinomas of the appendicular skeleton.

### Origin

In the past there has been much speculation about the origin of adamantinoma. Several hypotheses have been proposed for its histogenesis [4,6-22] (Table 1). Fischer [4] has suggested congenital implantation of epithelial cells whilst Ryrie [6] and Dockerty and Meyerding [7] favoured traumatic implantation. These suggestions have been criticized by Lederer and Sinclair [9], and Naji [10], who suggested a synovial origin. Although vascularity is not a striking feature of this tumor, at times these tumors can be very vascular, and it is the vascularity which is responsible for the fact that some authors regard these tumors as angioblastomas [11-14]. The etiology of the tumors is still a matter of debate but the most widely adopted theory is that of displacement of basal epithelium of skin during embryological development and is supported by the predominant involvement of anterior tibia, where enchondrally formed bone is closest to the skin surface [2]. It seems to suggest that adamantinoma is a tumor of epithelial origin. Based on ultrastructural and immunohistochemical studies, the tumor cells show strong positive staining with pan-cytokeratin antibody immunohistochemically [19] and by electron microscopy the cells have epithelial characteristics such as basal lamina, desmosomes, gap junctions, epithelial specific keratin and extracellular composition similar to epithelial tissue [23]. The possible relationship of adamantinoma to osteofibrous dysplasia is the subject of conflicting discussions and the potential link has implications for the diagnosis, prognosis, and treatment [2,24].

**Table 1:** Theories of origin of Adamantinoma

Theory	Authors, year
1. Fetal crest	Fischer, 1913 [4]
2. Basal cell lineage, Trauma	Ryrie, 1932 [6]
3. Implantation	Dockerty and Meyerding, 1942 [7]
4. Unknown	Baker et al, 1954 [8]
5. Synovial cell like	Lederer and Sinclair, 1954 [9], Naji et al 1964 [10]
6. Angioblastic	Changus et al, 1957, Elliot 1962, Llombart bosch and Ortuno-pacheco, 1978, Reed, 1982 [11-14]
7. Mesenchymal	Vinogradova, 1969 [15]
8. Dermal inclusion	Lichtenstein, 1977 [16]
9. Epithelial cell	Jaffe, 1958, Saacebra et al, 1968, Rosai and Pincus,1982, Ishida et al,1992, Hazelbag et al, 1993, Jundt et al, 1995 [17-22]

### Clinical features

Adamantinoma mostly occurs in the second to fifth decade. The median patient age is 25 to 35 years, with a range from 2 years to 86 years. It is slightly more common in men than women, with a ratio of 5:4 [25]. It rarely occurs in children with only a total of 119 pediatric patients, 65 boys and 54 girls reported in the medical literature searched in Pubmed-listed journals and Google [1,26].

The tumor has a striking predilection for the long bones (97 percent of cases) and, specifically, the tibia (80 to 85 percent of cases), represents the most characteristic clinical feature of this tumor. In 10 to15%, the lesion is also found in the ipsilateral fibula [27]. Statistically, the tumor remains unusually prevalent in the tibia, but all other major limb bones have been involved, and involvement of several short bones rarely has been reported [28]. Other bones that are involved, in order of decreasing frequency, include the humerus [29], ulna [30], femur [31], fibula [8], radius [32], innominate bones [33], ribs [34], spine[35] and rarely small bones of the hand and foot [36]. There have been only six cases of spinal adamantinoma reported [35]. There are extremely rare reports of adamantinomas arising exclusively from the pretibial soft tissues without any bony involvement [37,38].

The initial symptoms of adamantinoma are often indolent and nonspecific and depend on location and extent of the disease. The onset is insidious and its course shows a slow, progressive character. The patient often tolerates symptoms for many years before seeking medical attention. A history of significant trauma has been noted in about 60% of 200 cases reviewed by Moon and Mori [25]. Most patients present with swelling with or without pain. The patient may presents with bowing deformity of the tibia due to involvement of anterior tibial surface. Pathological fracture may be present in as many as 23% of the patients [39]. There have been two case reports of paraneoplastic hypercalcemia associated with tibial adamantinoma and pulmonary metastasis [40,41]. Spinal lesions may be manifested by neurologic symptoms in addition to pain [35].

### Radiology

Radiographically adamantinoma of bone displays features that are somewhat characteristic and help in establishment of presumptive diagnosis because of tumor's classic location and appearance. In the tibia, it usually appears as a central or eccentric, multilocular because of the sclerotic margins of overlapping radiolucencies, slightly expansile, sharply or poorly delineated osteolytic lesion. As previously stated, in long bones it is found in the diaphyseal location although metaphyseal extension of lesions or isolated involvement of a metaphysis is seen occasionally. The metaphyseal involvement makes the diagnosis more challenging because other tumors have to be considered in the differential diagnosis. The lesions are well-circumscribed involving anterior tibial cortex, with septations and peripheral sclerosis. Multifocality in the same bone is regularly observed. These multifocal radiolucencies surrounded by ring-shaped densities producing the characteristic "soap-bubble" appearance. The lesion is commonly intra-cortical, but may destroy cortex and invade the extracortical soft tissues in about 15% of cases [1]. The entire lesion may have a prominent sclerotic margin indicative of slow growth. The periosteal reaction is variable from minimal to prominent [23].

Radiologically, the differential diagnosis of adamantinoma should include osteofibrous dysplasia and fibrous dysplasia. Distinguishing osteofibrous dysplasia and fibrous dysplasia from adamantinoma may be difficult; however, diaphyseal location, involvement of the anterior cortical bone with extension toward the bone marrow, single or multiple nodular lesions in one or more foci, and intense and homogeneous enhancement may contribute to the diagnosis of adamantinoma. OFD is specifically located in the cortex without involvement of the medullary canal [42].

Computed Tomography (CT) and Magnetic Resonance Imaging (MRI) have been used to study the lesions of adamantinoma and usually the findings are not specific. CT scan shows the cortical involvement and the soft tissue extension when it exist. However, does not show the intraosseous extension of the tumour. CT does play a role in detecting pulmonary metastases and as such plays a role in the routine work-up [1].

MRI is pivotal for precise locoregional staging, especially for depiction of distant cortical foci, soft tissue, and intramedullary extension and thus is useful for determining tumor-free margins and strategies for reconstructive surgery. Van der Woude et al described two morphological patterns on MR imaging: a solitary lobulated focus versus a pattern of multiple small nodules in one or more foci. If MRI scanning is used to study adamantinomas, the tumors demonstrate low signal intensity on T1-weighted spin echo images and high signal on T2-weighted images. Because these appearances are also typical of most tumors, these findings are nonspecific [43].

Use of nuclear medicine to study adamantinomas is a relatively new undertaking. The following findings correspond to adamantinomatous lesions: increased blood flow in the region of the tumor, increased blood pooling, and increased accumulation of technetium-99m methylene diphosphate in the area of the tumor. In addition, bone scan may show a coexisting fibular involvement [44].

### Pathology

Histologically, the classic adamantinoma is a biphasic tumor and characterized by epithelial and osteofibrous components that may be intermingled with each other in various proportions and differentiating patterns. Adamantinoma is classified into 2 distinct types: classic and differentiated. Classic adamantinoma usually occurs in patients older than 20 years, whereas differentiated adamantinoma (Osteofibrous dysplasia (OFD) – like/regressive/juvenile/intracortical adamantinoma) occurs almost exclusively in patients younger than 20 years [45]. In addition, the two subtypes of adamantinoma have distinct radiographic and histologic differences (Table 2).

**Table 2:** Classification of Adamantinoma

Features	Classic	Differentiated
Age	More than 20 years, Adults	Less than 20 years, Children
Radiology	Soft tissue or intramedullary involvement regularly observed	Intra cortical location indistinguishable from OFD
Histopathology	Admixture of both epithelial and osteofibrous component, most commonly solid nests of basaloid cells	OFD like pattern lacks conspicuous nests and masses of epithelial cells. Scattered positivity of epithelial elements for cytokeratin
Behavior	Aggressive clinical course	Relatively benign

Classic adamantinoma is in general characterized by admixture of both epithelial and osteofibrous components that are associated with various proportions and differentiation patterns. OFD-like adamantinoma is characterized by predominance of osteofibrous tissues, in which small groups of epithelial cells are only detected by careful search or immunohistochemistry.

#### Gross appearance

The appearance varies but most often the tumor is yellow gray or grayish white and fleshy or firm in consistency. Some OFD like adamantinomas are more solid because of the presence of relatively large bone forming areas. Occasionally tumors show macroscopic cysts range from a few millimeters to centimeters in diameter. These cystic spaces contain a straw colored or blood like fluid on gross examination [46].

### Microscopic examination

**Figure 1:**
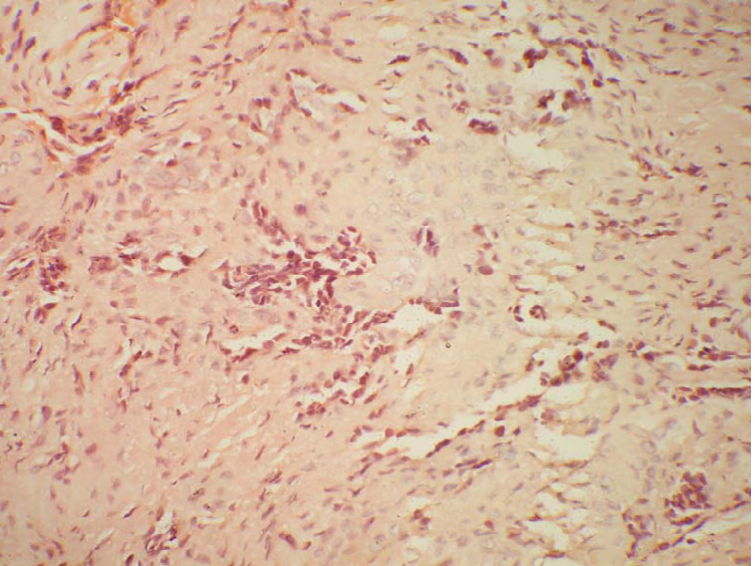
**An adult female presented with pain in the right lower leg.** On imaging, a cortical lytic lesion was seen in the lower end of tibia. Photomicrograph to show solid nests of basaloid cells embedded in a fibrous stroma H & E × 400.

**Figure 2:**
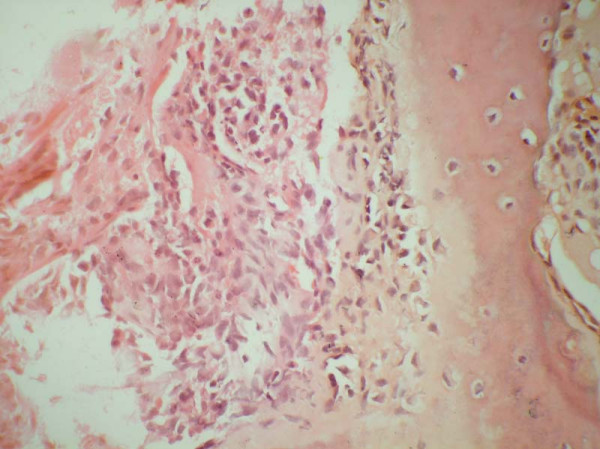
Higher magnification to show hyperchromatic cells with moderate nuclear pleomorphism H & E × 600.

The neoplastic cells of the classic adamantinoma range from small to large in size with finely dispersed chromatin and an overall bland appearance. A few cases from a large series have exhibited nuclear atypia. Mitotic figures are usually infrequent, most reporting 0–2 mitoses per 10 high power fields [27,47]. Several patterns of growth have been described such as tubular, basaloid, squamous, spindle-cell, and osteofibrous dysplasia-like variant. In classic adamantinomas, the most common form consists of solid nests of basaloid cells (Fig 1 &2). Tubular adamantinomas are consisted of narrow cords of epithelial cells with central discohesion resulting in a vascular or glandular appearance. In the basaloid variant, the epithelial cells exhibit solid nests of basaloid cells with distinctive peripheral palisading. The squamous variant with or without keratinization may resemble well differentiated squamous cell carcinoma. The spindled form shows uniform spindling with presence of clefts lined by epithelial cells. The histologic features of differentiated adamantinoma are predominantly characterized by a pattern of osteofibrous dysplasia [45].

Foci of calcification [48], giant cells [8], xanthoma and spindle cells [49] have all been also described in adamantinoma. Sarcomatous dedifferentiation of the epithelial component has been described by Hazelbag et al [50]. In addition a rhabdoid variant of differentiated adamantinoma has been reported by Povysil et al [51].

#### Relationship between adamantinoma and OFD

It has been postulated that the predominance of an osteofibrous dysplasia-like pattern in differentiated adamantinoma is the result of a secondary reparative process overgrowing matured and regressing tumor tissue. It is possible that this process may lead to the total elimination of recognizable tumor cells from the lesion. Therefore, osteofibrous dysplasia, which has a similar anatomic location, age distribution, and radiologic appearance as differentiated adamantinoma, may, in some cases, represent the evolution of an underlying adamantinoma. Analysis suggests that long bone adamantinoma could be another member of the unique family of tumors that may regress spontaneously [45].

Because some osteofibrous dysplasia-like tumors have progressed to classic adamantinomas, it is hypothesized that the former is a potential precursor of the latter, showing mesenchymal-to-epithelial transformation. There are two cases reported in the literature of progression from differentiated to the classic type [47]. There have been controversies as to the potential correlation among classic adamantinoma, osteofibrous dysplasia-like adamantinoma and osteofibrous dysplasia, all of which originate mainly in the tibial cortex and have close radiographic and histological similarities.

A probable histogenetic relationship exists between the three entities. Indeed, the only apparent difference between osteofibrous dysplasia and differentiated adamantinoma is the presence of an epithelial component readily identifiable on routine hematoxylin and eosin stained sections and their identification only by immunohistochemical means in about 80% of osteofibrous dysplasia lesions (Table 3).

**Table 3:** Differences between Osteofibrous dysplasia and Adamantinoma

Features	Osteofibrous dysplasia	Adamantinoma
Nature	Benign condition	Locally aggressive
Age	Less than 10 years	2 year to 86 years
Site	May involve both tibia and fibula.	90% tibial involvement, In 10–15% cases, ipsilateral fibular involvement, Rarely pretibial soft tissue and other bones
Clinical presentation	• Pain, swelling, pseudoarthrosis, bowing, pathological fracture may occur	• With or without Pain, swelling, pathological fracture in 25% cases
	• seldom progresses during childhood, and any progression of the lesion stops after puberty	• progressive during adult age
Predisposing history of trauma	Absent	Present
Radiology	Periosteal reaction present	Periosteal reaction is variable
	Intra cortical	15% of cases, there is extracortical extension into soft tissues
	Limited to anterior cortex	Single or multiple nodular lesions in one or more foci in medulla
	Well marginated with marginal sclerosis, ground glass appearance	Sharply or poorly delineated osteolytic lesion. with septations and peripheral sclerosis, characteristic "soap-bubble" appearance
Histopathology	Zonal phenomenon present Scattered epithelial cells recognized on IHC	Absent Presence of epithelial cells forming small nests/strands recognized in H&E
Recurrence	Local recurrence in 25%.	Tends to recur in 18–32%
Metastasis	No metastasis	Metastases may occur in 15–30%, Lung and Lymph nodes usually involved
Regression	Spontaneous regression at puberty in 33% cases	Regression ± [45]

Czerniak et al [45] proposed the existence of a continuum of fibro-osseous lesions with osteofibrous dysplasia at one end of the spectrum, classic adamantinoma at the other, and differentiated adamantinoma intermediately. This hypothesis is also supported by other authors [52,53]. Mirra [2] considered this possible relationship by calling osteofibrous dysplasia "juvenile adamantinoma". This concept, however does not rule out the possible existence of *de novo *osteofibrous dysplasia not related to adamantinoma. Sweet et al [54] studied 30 cases of osteofibrous dysplasia to determine whether it is a precursor lesion to adamantinoma. They concluded that there was no conclusive evidence of a precursor role for osteofibrous dysplasia. However cytogenetic studies have demonstrated similar alterations in the two lesions [55].

### Relationship between adamantinoma and Ewing's sarcoma

Recently, the least common variant of adamatinoma has been described as Ewing's-like adamantinoma or adamantinoma-like Ewing's. The variant is characterized by anastomosing cords of small, uniform, round cells set in a myxoid stroma. These cells exhibit features of both epithelial cells and neuroendocrine cells on ultrastructural examination. Immunohistochemical studies have shown the tumor cells to contain both epithelial and neural antigens including the Ewing's sarcoma-related antigen [56,57]. Bridge et al [52] documented 11;22 translocation in the nuclei of cytokeratin-immunoreactive cells, and therefore considered the tumors to be variants of Ewing's sarcoma rather than Ewing-like adamantinoma. They termed the lesion "adamantinoma-like Ewing's sarcoma". In contrast, Hauben et al. reported that 12 cases of adamantinoma lacked both EWS-FLI-1 and EWS-ERG fusion transcripts [58]. Folpe et al. described three cases of "adamantinoma-like" Ewing's sarcoma showed a distinctly nested, epithelioid growth pattern with surrounding desmoplasia, and characteristically expressed high molecular-weight cytokeratins and pan-cytokeratins with genetic confirmation [59]. Recently Fujii et al reported a case of adamantinoma-like Ewing's sarcoma with EWS-FLI1 fusion gene [60]. The histogenesis of this lesion is not clear; however, Ewing's sarcoma is considered to be derived from primitive, pluripotential stem cells that may differentiate into cells with mesenchymal, epithelial, and neuronal features. This may result in expression of phenotypes showing both epithelial and neuroectodermal differentiation in Ewing's sarcoma. These data indicate that tumors showing overlapping morphological and immunohistochemical features can be readily distinguished with molecular techniques. Appropriate treatment of this lesion has not yet been established, but standard pre- and postoperative chemotherapy and wide surgical excision for Ewing's sarcoma has been recommended. Recently, the risk-adapted treatment strategy including high dose chemotherapy (HDCT) with peripheral blood stem cells transplantation (PBSCT) has been proposed [61]. However, previous studies demonstrated that HDCT with PBSCT in the treatment of Ewing's sarcoma have limited benefits [62,63]. Further study will be needed to clarify the differences of prognosis and chemosensitivity between conventional Ewing's sarcoma and "adamantinoma-like Ewing's sarcoma".

### Immunohistochemistry

**Figure 3:**
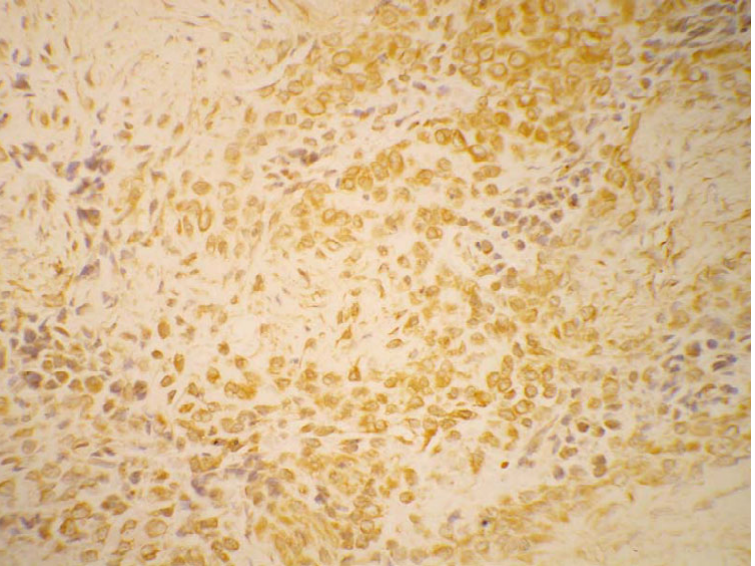
Immunohistochemistry for cytokeratin shows strong positivity with in the tumor cells × 600.

Regardless of histologic subtypes, all adamantinomas have been uniformly positive for keratins 14 and 19 [21]. Immunohistochemically, the epithelial cells show coexpression of keratin, especially basal epithelial cell keratins (CKs 5, 14 and 19) and vimentin [64] (Fig 3). The keratin immunoreactivity pattern is independent of histologic subtype, despite marked variety in differentiation pattern, suggesting a common histogenesis for all subtypes of adamantinoma. Furthermore, the pattern is conserved both in local recurrences and in metastasis. The pattern differs significantly from other bone and soft tissue tumors with known epithelial characteristics, e.g., synovial sarcomas, chordomas, and epithelioid sarcomas, in that it lacks immunoreactivity of keratins 8 and 18. Therefore a basal epithelial cell-like differentiation of adamantinomas is suggested [21].

There is still debate as to whether the fibrous part should be designed as a benign neoplastic element of a biphasic tumour or as a reactive non-neoplastic tissue next to an epithelioid bone tumour. Bovee et al [65] examined the relationship between the epithelial and the stromal components of adamantinomas by comparing the immunohistochemical expression of the proliferation marker Ki-67, epidermal growth factor and epidermal growth factor receptor and fibroblast growth factor type 2 in the two cell populations. These antigenic factors were present either exclusively or predominantly in the epithelial component, suggesting that the epithelial component constitutes the primary proliferating neoplastic cell population that is able to stimulate a reactive fibrous growth. Additional studies by Hazelbag et al [66] utilizing DNA flow cytometry and p53 immunohistochemistry demonstrated aneuploidy and significant p53 immunoreactivity only in nuclei of cells of epithelial phenotype. Furthermore, in several cases with pulmonary metastasis, only cytokeratin-positive epithelial cells and not the osteofibrous stromal component were detected in the metastatic lesions. These results suggested that either adamantinoma consists of a malignant epithelial part with a reactive osteofibrous stroma or that the malignant epithelial cells develop next to a proliferating benign fibrous component.

Maki and Athanasou [67] correlated proto-oncogene product and matrix protein expression in osteofibrous dysplasia and adamantinoma. They found C-fos and c-jun, expression in OFD and in the fibrous and epithelial components of differentiated and classical adamantinomas. Staining for collagen IV, laminin and galectin-3, was seen in OFD and around cell nests in adamantinoma. However E-, P-, and N-cadherin expression was found only in classical adamantinoma. Osteonectin was detected in both the epithelial and fibrous components of adamantinomas, but osteopontin and osteocalcin were not seen in classical adamantinomas. The results show common expression of a number of oncoproteins and bone matrix proteins in adamantinoma and OFD.

### Cytogenetics

Cytogenetic analysis has contributed greatly to the understanding of the histopathogenesis of adamantinoma and osteofibrous dysplasia. Unfortunately, because of their rarity, cytogenetic reports concerning these tumors are few. Review of previously reported cases reveal extra copies of chromosomes 7, 8, 12, 19, and/or 21 in classic and differentiated adamantinoma [55,68-70]. Extra copies of one or more of these same chromosomes with the exception of chromosome 19 have also been seen in osteofibrous dysplasia, lending further support to an osteofibrous dysplasia/adamantinoma relationship. Various studies related to cytogenetic analysis in adamantinoma are described in Table 4.

**Table 4:** Cytogenetic findings in Adamantinoma

Author	Type of adamantinoma	Karyotype
Mandahl et al [68]	Classic	46,XX,t(1;13;22), t(15;17)
Sozzi et al [69]	Classic	52,XY +7,+12,+13,+19,+der(7), +der(13)
Hazelbag et al [70]	Classic	50,XY,+7,+8,+12,+19
	Classic	50,XY,+7,+8, +12,+19
	Classic	51,XY,+X,+7,+12,+19,+21/50,
	Differentiated	48,XY,+7,+8
	Differentiated	46,XX,t(2;11), inv(11)
Kanamori et al [55]	Classic	54,XX,-1,+5,+der(7),+der(8), +der(9),+19,+20,+21,+mar1,+mar2
	Classic	7,8,12,19 extra copies
	Classic	7,8,12,19 extra copies
	Differentiated	54,XY,+5,+7,+8,+12,+12,+14,+19,+21/53

### Cytopathology

There are several publications that confer the radiologic, clinical, and histopathologic features of adamantinoma; on the contrary, only few cases of the cytologic findings of adamantinoma are found [71-76]. Cytologic features of adamantinoma in various studies have been reviewed and described in Table 5.

**Table 5:** Cytopathology of Adamantinoma

Author	Age of patient	Site of tumor	Behavior of tumor	Cytologic features
Hales and Ferrell [71]	NA	Tibia	Primary	Peripheral palisading, and prominent extracellular hyaline-like material
Galera-Davidson, et al [72]	31	Tibia	Recurrent	Single-lying cells, small clusters and Indian files, with some nuclear molding. Three cell types (1) large polygonal cells with chromatin clearing, (2) smaller cells with poorly defined cytoplasm and dense chromatin and (3) fusiform cells
Laucirica, et al [73]	25/M	Tibia	Primary	Numerous sheets and single cytologically bland spindle cells with "chromatinic membrane folds"
Perez-Ordonez and Bedard [74]	Two cases	Lung	Metastatic	Small round and spindle cells with indistinct cytoplasm. The nuclei had delicate nuclear membranes, with finely dispersed chromatin and occasional micronucleoli. No pleomorphism.
Tabei, et al [75]	One case	Lung	Metastatic	Clusters of small cells with either prominent nucleoli or spindle-shaped hyperchromatic nuclei.
Flowers et al [76]	One case, 32/M	Tibia, Lung	Recurrent and metastatic	Numerous nuclear membrane grooves within the polygonal cells

NA: Not available

### Differential diagnosis

Because of its rarity and differing clinicoradiologic and histological patterns, adamantinoma may resemble numerous conditions (Table 6).

**Table 6:** Differential diagnosis of Adamantinoma

**Lesions**	**Age (yrs)**	**Sex M:F**	**Site**	**Location**	**Clinical Features**	**Radiological Features**	**Gross**	**Microscopy**	**IHC**	**Treatment**	**Remarks**
**Aneurrysmal bone cyst**	10–15	1:1	Vertebrae, flat bones, humerus, tibia	Metaphysis	Usually history of trauma, f/b gradually increasing swelling with little pain. There may be pathological fracture or spinal pressure symptoms	Well defined radiolucent, eccentric cyst	Spongy hemorrhagic mass	Fibrous tissue, vascular spaces	IGF-1	Curettage with bone grafting	May heal spontaneously Benign
**Unicamaral bone cyst**	10–20	3:1	Humerus, femur	Metaphysis	Usually asymptomatic	Well demarcated, radiolucent cyst extending up to physeal plate	Cystic mass	Well vascularised fibrous tissue with hemosiderin and cholesterol clefts	NA	Curettage with bone grafting	Benign
**Fibrous dysplasia**	10–30	3:2	Neck of femur, tibia, base of skull	Metaphysis diaphysis	May be mono or polyostotic, pathological fractures and progressive deformity	Cystic areas in metaphysis, lucent patches typically have ground glass appearance	Coarse gritty, Grayish yellow	Loose cellular fibrous tissue with widespread patches of woven bone and scattered giant cells	NA	Depending upon location and type of deformity	Locally aggressive, rarely sarcoma Develops, Associated Albright syndrome
**Chondromyxoid Fibroma**	10–25	1:1	Tibia, fibula, femur, feet, pelvis	Metaphysis	Asymptomatic, pathological fracture	Eccentrically placed lytic lesion with well defined sclerotic margins	Solid yellowish white or tan	Patches of myxomatous tissue with stellate cells, islands of hyaline cartilage, fibrous tissue	S-100	Excision	Benign
**Giant cell tumor**	20–40	4:5	Epiphysis and metaphysis	Femur, tibia, radius	Pain with swelling, pathological fracture	Eccentric, cystic lesion in mature bone, extending up to the subchondral plate, soap bubble appearance	Reddish fleshy mass	Multinucleated giant cells, stromal cells, cellular atypia with mitotic figures	Muscle actin, alpha-smooth muscle actin and CD68	Depending upon severity of lesion, curettage with bone graft to excision	Potentially malignant, 50% recur, 10% metastasize
**Eosinophilic granuloma**	5–10	2:1	Metaphysis	Flat bones, mandible, spine and long bones	Local pain, swelling and tenderness	Well demarcated oval radiolucent area, associated with marked reactive sclerosis	Soft, granular or gelatinous mass	Sheets of Langerhan's cells	CD-1a, S-100	Excision or curettage	Usually heals spontaneously
**Osteomyelitis**	Any age	Male outnumbers female	Metaphysis, diaphysis	Distal femur, proximal tibia, proximal femur and proximal humerus	Discharging sinus, fever, malaise, local pain and swelling	Multiple aggressive lytic lesions, serpiginous lytic pattern is more specific sequestrum and involucrum are of**t**en seen	Bone destruction, cavities containing pus with sequestrum	Inflammatory cells around areas of acellular bone or microscopic sequestra, prominent periosteal bone proliferation	NA	Removal of sequestrum antibiotic, excision of sinuses	Variable prognosis
**Chondrosarcoma**	30–60	3:1	Metaphysis	Pelvis, rib, humerus, femur, vertebrae	Dull ache or gradullay enlarging lump	Radiolucent area with central flecks of calcification	Lobulated with gelatinous shiny areas	Lobules of highly atypical cells, including binucleate cells.	S-100, Vimentin	Wide excision	Malignant
**Epithelial metastasis**	Any age	Variable	Pattern of these lesions is more diffuse than regional	Vertebrae, pelvis, rib, femur, skull, humerus(rare below elbow and knees)	Pain	Bone destruction, osteolytic; osteoblastic response with Ca prostate	Osteolytic, rarely sclerotic	Malignant cells with vascular invasion	Depend on the site of primary	Osteoclast inhibiting agents, radiation therapy	Most common primaries breast, prostate, lung, kidney, and thyroid
**Hemangioendothelioma**	20–30	2:1	Metaphyseal, diaphyseal, or, less commonly, epiphyseal.	Calvarium, femur, tibia and feet	Pain and swelling	Expansive, osteolytic and poorly demarcated lesions. "soap-bubble" matrix with a sclerotic margin	Well-circumscribed, irregular borders soft, bright red hemorrhagic appearance	Solid nests and anastomosing cords of round, polygonal, or spindle-shaped cells with eosinophilic cytoplasm. Intracytoplasmic vacuolization	Factor VIII, CD31, CD34	Depending on the grade of the lesion currettage, or wide resection	Multifocal in up to 50% of cases and may be mono-ostotic or polyostotic locally aggressive, metastasize to bone and lung
**Angiosarcoma**	Any age	Older male	Metaphyseal and diaphyseal	Any bone, multifocal	Pain and swelling	Eccentric, lytic, metaphyseal and diaphyseal, well circumscribed areas of rarefaction	Variable	Anastomosing vascular channels lined by highly atypical endothelial cells	Factor V11I, CD31, CD34	Wide resection and adjuvant therapy	Malignant
**Nonossifying fibromas**	10–20	1:1	Tibia, femur	Metaphysis	Pain	Eccentric, sharply delimited lesion	Solid, Granular, brown, dark red	Fibrous tissue arranged in storiform pattern, foamy and hemosiderin laden macrophages	Little or no application	Not necessary	Benign

ABBREVIATION: IGF: Insulin like growth factor, IHC: Immunohistochemistry, f/b: followed by, NA: Not applicable

### Behavior

Adamantinomas are locally aggressive tumor and are extremely slow growing with the potential to metastasize. Recurrence of tumor is frequent after inadequate therapy, and the behavior of the recurrent neoplasm resembles more and more that of a sarcoma. This low-grade, slowly-growing malignancy metastasizes in about 15–30 percent of cases by both hematogenous and lymphatic routes to other parts of the body, usually to the lungs or nearby lymph nodes; bone and abdominal viscera make up a minority [25,27,65,77]. Although exhibiting varying histological patterns, no correlation between histology and clinical course was seen. However, Theros and Ishak [78] reported that the more malignant the lesion is, the less distinctive the honeycomb pattern. Some investigators think that the Ewing's sarcoma-like variant of conventional adamantinoma, is accompanied by more aggressive behavior and a poorer prognosis [56]. In addition intra-lesional treatment, male patient, pain at presentation, short duration of symptoms, young age (less than 20 years) and lack of squamous differentiation of tumor seem to influence an unfavorable clinical outcome [77].

Local recurrence rate varies from 18–32% [27,65]. Although ascertaining accurate mortality statistics is difficult because of the extremely rare nature of this tumor, mortality rates of 13% to 18% have been reported [25,27].

### Treatment

Current treatment of adamantinoma, including en bloc tumor resection with wide operative margins with limb reconstruction and limb salvage, provides lower rates of local recurrence than has been previously reported [27]. Quereshi et al reported in a review of 70 patients, en bloc tumor resection with wide margins and limb salvage was shown to have 10 year survival rate of 87.2% [39]. Amputation for adamantinoma has not been shown to improve survival when compared with the limb preserving surgery but may be advisable if local recurrence occur or if en-bloc resection and limb salvage is not an option. The limb reconstruction can be performed with distraction osteogenesis, allografts, vascularised fibular autografts (preferred) and metallic segmental replacement [23]. Unfortunately neither radiation therapy nor chemotherapy has been proven effective in the treatment of this insidious tumor [28].

## Conclusion

Although the incidence of adamantinoma is low, it is important to recognize this rare bone tumour, since in early stages of the disease adequate treatment will result in an excellent prognosis. The histologic features of primary adamantinoma are usually characteristic enough for a presumptive diagnosis; however, the rarity and the heterogeneity of the tumor could pose diagnostic uncertainty in some cases, especially those arising in non-tibial locations. Extensive sampling of the lesion is important, especially in the differentiated adamantinoma where the epithelial component may be seen only focally. To assure the histological diagnosis pathologists should employ immunohistochemistry for demonstrating the sometimes sparse epithelial cell nests when radiology is suggestive for adamantinoma. Correct diagnosis should lead to resection with wide surgical margins.

## Competing interests

The author(s) declare that they have no competing interests.

## Authors' contributions

DJ and VKJ are primarily responsible for drafting, literature search, and submission of the manuscript. RKV established the diagnosis of the case described in figures. PR and YK provided images. All authors have read and approved the final manuscript.
